# miR-34 and p53: New Insights into a Complex Functional Relationship

**DOI:** 10.1371/journal.pone.0132767

**Published:** 2015-07-15

**Authors:** Francisco Navarro, Judy Lieberman

**Affiliations:** 1 Cellular and Molecular Medicine Program, Boston Children’s Hospital, Boston, Massachusetts, United States of America; 2 Department of Pediatrics, Harvard Medical School, Boston, Massachusetts, United States of America; IRCCS-Policlinico San Donato, ITALY

## Abstract

miR-34, a tumor suppressor miRNA family transcriptionally activated by p53, is considered a critical mediator of p53 function. However, knockout of the mouse miR-34 family has little or no effect on the p53 response. The relative contribution of different miR-34 family members to p53 function or how much p53 relies on miR-34 in human cells is unclear. Here we show that miR-34a has a complex effect on the p53 response in human cells. In HCT116 cells miR-34a overexpression enhances p53 transcriptional activity, but the closely related family members, miR-34b and miR-34c, even when over-expressed, have little effect. Both *TP53* itself and *MDM4*, a strong p53 transactivation inhibitor, are direct targets of miR-34a. The genes regulated by miR-34a also include four other post-translational inhibitors of p53. miR-34a overexpression leads to variable effects on p53 levels in p53-sufficient human cancer cell lines. In HCT116, miR-34a overexpression increases p53 protein levels and stability. About a quarter of all mRNAs that participate in the human p53 network bind to biotinylated miR-34a, suggesting that many are direct miR-34a targets. However, only about a fifth of the mRNAs that bind to miR-34a also bind to miR-34b or miR-34c. Two human cell lines knocked out for miR-34a have unimpaired p53-mediated responses to genotoxic stress, like mouse cells. The complex positive and negative effects of miR-34 on the p53 network suggest that rather than simply promoting the p53 response, miR-34a might act at a systems level to stabilize the robustness of the p53 response to genotoxic stress.

## Introduction

Genotoxic stress activates p53 transcription of many genes, leading to cell cycle arrest, apoptosis or senescence [1]. Multiple miRNAs, including the miR-34 family, are transcriptionally activated by p53. In turn miRNAs regulate expression of many p53-induced genes [2, 3]. The miR-34 family consists of 3 miRNAs—miR-34a on human chromosome 1p36 and miR-34b/c, co-transcribed on human chromosome 11q23. In mice, miR-34a is expressed in most tissues, while miR-34b/c are predominantly expressed in lung and testis [4, 5]. Three chromosome 5q11.2 miRNAs (miR-449a/b/c) share a seed sequence with miR-34, and have a tissue distribution similar to that of miR-34b/c [6, 7]. miR-34a is not expressed in many cancers because of chromosomal deletion or promoter methylation [8]. Ectopic expression of miR-34a leads to cell cycle arrest, apoptosis or senescence, mimicking p53 activation [9]. Although multiple miR-34a targets have been identified, it is not clear which targets determine miR-34a’s contribution to the p53 response. More importantly, it is unclear how much miR-34a contributes to p53 function. Although antagonizing miR-34a in human cells impairs p53 function in a few studies [4, 10, 11], mice genetically deficient in all miR-34 family genes have unimpaired stress responses [12].

We set out to study how the different miR-34 miRNAs contribute to p53 function, analyze whether they regulate overlapping sets of targets and determine if miR-34 is essential for p53-mediated function in human cells. Here, we provide evidence showing that, despite sharing an identical seed sequence, miR-34b/c do not enhance p53 transcriptional activity and they regulate non-overlapping genes, involved in distinct biological processes. We also show that although miR-34a can have a positive effect in p53 transcriptional activity and protein stability, by targeting multiple p53 inhibitor genes (*MDM4*, *SIRT1*, *MTA2*, *HDAC1*, *YY1*), as has been previously described [11, 13–15], it can also have a negative effect on TP53, by directly targeting *TP53* mRNA. More importantly, we show that the net effect of miR-34a over-expression on TP53 levels is cellular context dependent. Last, using TALEN-generated (Transcription Activator-Like Effector Nucleases) HCT116 and MCF7 miR-34a knock-out cells, we show that the p53-mediated response to genotoxic stress is unimpaired in these cells. Thus, our results suggest that miR-34a is dispensable for the p53-mediated response to stress in human cells, as it is in mice [12].

In summary, although our data confirm the strong interplay of p53 and miR-34a, they suggest a complex functional relationship. Rather than simply promoting p53 function, miR-34a might act at a systems level to affect multiple genes in the p53 network, both positively and negatively. The net effect may be to stabilize and reinforce the p53 response.

## Results

### Ectopic expression of miR-34a, but not miR-34b/c, increases p53 transcriptional activity

**Fig 1:**
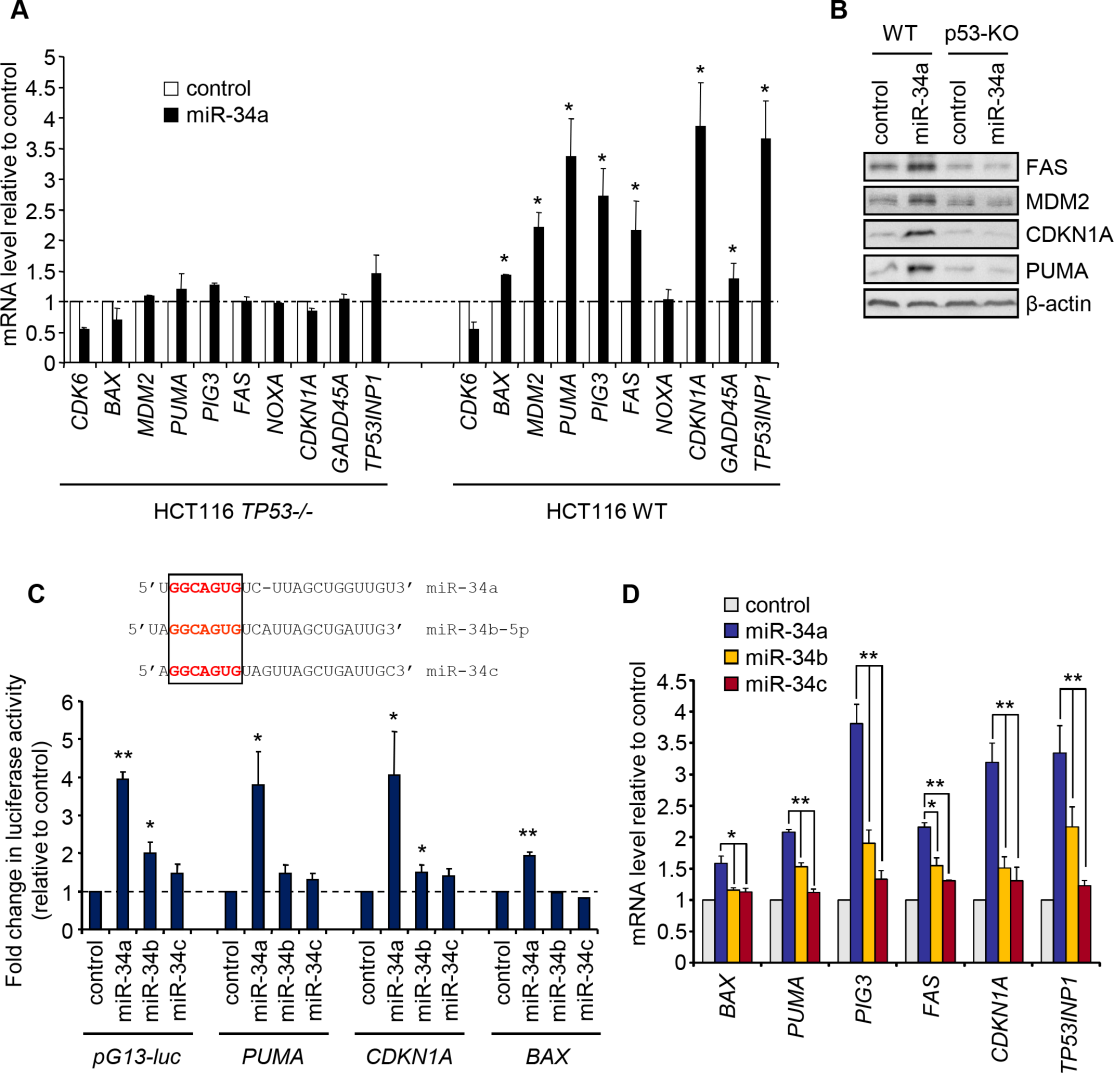
Overexpression of miR-34a, but not miR-34b/c, enhances p53 transcription in HCT116 cells. **(A)** qRT-PCR analysis of mRNA levels of p53 transcriptional targets, normalized to *GAPDH*, in miR-34a or cel-miR-67 (control) overexpressing WT and *TP53* ^*-/-*^ HCT116 cells. **(B)** Immunoblot showing protein levels of some p53 transcriptional targets. **(C)** Effect of miR-34 OE on *Firefly luciferase* reporters driven by the promoters of the p53 targets *PUMA*, *CDKN1A* and *BAX* or a tandem repeat of 13 p53 binding consensus sites (pG13-luc). Normalized Firefly luciferase activity, relative to *Renilla* luciferase activity, after miR-34 transfection is plotted as fold change relative to control miRNA-transfected sample. Alignment of the miR-34 family with the seed sequence highlighted in red is shown at top. **(D)** qRT-PCR analysis of p53 transcriptional target mRNAs after transfecting control or miR-34 mimics into HCT116 cells. Bar graphs show mean +/- SD of at least three independent experiments (*, p<0.05; **, p<0.01, relative to control miRNA-transfected cells, 2-tailed Student’s t-test).

To assess the effect of miR-34a on the p53 response, we used qRT-PCR to analyze the effect of miR-34a overexpression on 9 p53-activated gene mRNAs in wild type (WT) and *TP53* ^-/-^ HCT116 cells. miR-34a overexpression increased all but one of the targets tested (*BAX*, *MDM2*, *PUMA*, *PIG3*, *FAS*, *CDKN1A*, *GADD45A* and *TP53INP1*, but not *PMAIP1* (*NOXA*)), but only in WT cells (Fig 1A). As expected, miR-34a overexpression reduced expression of *CDK6*, a known miR-34a gene target, similarly in p53-sufficient and-deficient cells. The protein products of *FAS*, *MDM2*, *PUMA* and *CDKN1A* also increased, only in p53-sufficient cells (Fig 1B). We next used luciferase reporter promoter assays, in p53-sufficient HCT116 cells, to assess whether miR-34 overexpression enhanced promoter activities of a sequence of 13 tandem repeats of the p53 binding site (pG13-luc) [16] or the promoters of p53-regulated genes, *PUMA*, *CDKN1A* (the gene encoding p21/WAF1) and *BAX*. miR-34a overexpression increased by 4-fold the luciferase activity of the pG13-luc, *CDKN1A* and *PUMA* promoters and increased by 2-fold *BAX* promoter activity (Fig 1C). miR-34b-5p (hereafter designated miR-34b) overexpression had a modest, but significant, effect on 2 of the 4 promoters, while miR-34c did not significantly increase activity of any (Fig 1C), even though it was over-expressed more than a hundred fold above its endogenous level after genotoxic stress (data not shown). Consistent with this result, induction of 6 p53 transcriptional targets in HCT116 cells was significantly less after miR-34b or miR-34c overexpression than after miR-34a overexpression (Fig 1D), despite highly elevated miRNA overexpression (S1A Fig). Thus miR-34-mediated increased p53 transcription is largely limited to miR-34a.

### miR-34a and miR-34b/c regulate different biological processes

**Fig 2:**
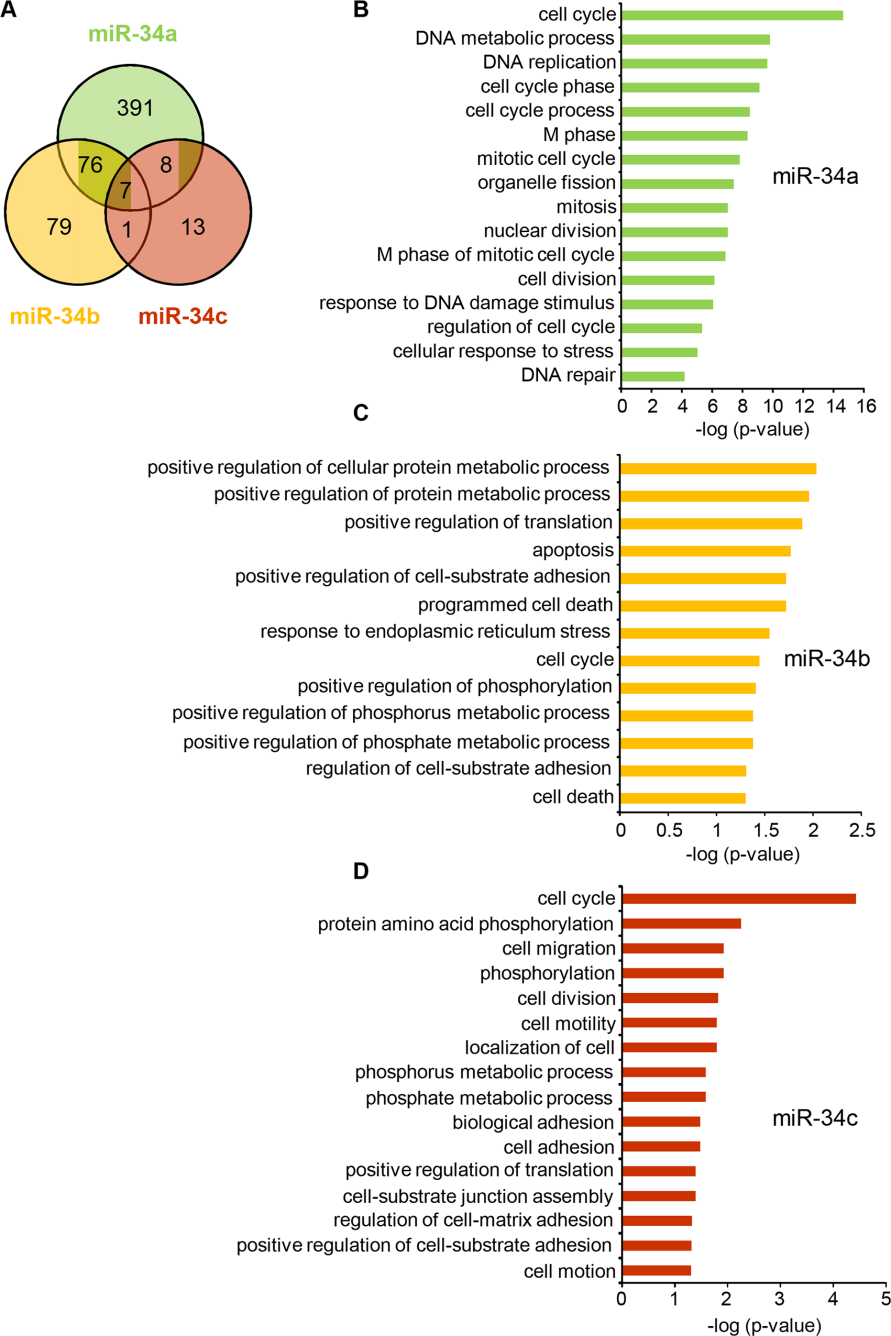
Genome-wide transcriptome analysis of miR-34 OE HCT116 cells. **(A)** Overlap of genes down-regulated ≥ 1.5 fold in miR-34 OE HCT116 cells compared to control-transfected cells. **(B-D)** Top enriched biological processes (p<0.05) of all down-regulated genes in miR-34 overexpressing HCT116 cells, as determined using the DAVID tool. A complete list of significantly enriched biological processes is provided in S2 Table.

Our observation that only miR-34a overexpression enhances p53-mediated transcription was surprising since the miR-34 family active strands are highly homologous—the seed (residues 2–9) and residues 11–17 and 19–21 are identical (Fig 1C). To determine whether the miR-34 family might regulate non-overlapping mRNAs, we performed gene microarray analysis of HCT116 cells overexpressing each family member (S1B Fig). 482, 163 and 29 mRNAs were significantly down-regulated (fold decrease ≥ 1.5 fold relative to miRNA control) after miR-34a, miR-34b or miR-34c overexpression, respectively (Fig 2A and S1 Table). About half the mRNAs down-regulated by miR-34b or miR-34c were also down-regulated by miR-34a, but less than a fifth (91 of 482) of the genes down-regulated after miR-34a overexpression were down-regulated by miR-34b or miR-34c (Fig 2A), suggesting that individual miR-34 miRNAs regulate unique targets. To assess whether regulation of these unique targets might translate into different biological functions, we performed a Gene Ontology (GO) analysis of the down-regulated genes using DAVID [17, 18]. Not unexpectedly, miR-34a-regulated genes were over-represented in genes that regulate the cell cycle, mitosis and cell division, DNA metabolism/replication/repair and the response to stress and DNA damage (Fig 2B). Although miR-34b/c suppressed genes were also enriched for involvement in the cell cycle, most of the over-represented processes of the miR-34b/c suppressed genes had non-overlapping functions in protein metabolism/translation, cell adhesion/motility/migration, and apoptosis/cell death (Fig 2C and 2D), some of which are related to impaired development of ciliated tissues seen in KO mice [6, 7]. These data together suggest that miR-34a and miR-34b/c serve different biological functions. In particular, the effect on p53 is predominantly mediated by miR-34a.

### miR-34a targets many p53 network genes

We previously used streptavidin pull-downs (PD) of Bi-miR-34a-transfected HCT116 and K562 cells to identify miR-34a-regulated genes [19]. p53 response gene mRNAs were significantly enriched by miR-34a PD in both cell lines. To examine miR-34a’s role in regulating p53 function, we analyzed how many annotated p53 network genes (S3 Table) were enriched in the Bi-miR-34a PD in HCT116 cells. These genes, compiled using the p53Knowledgebase (http://p53.bii.a-star.edu.sg/index.php), encode for p53 transcriptional targets, human p53-interacting proteins, or transcription factors that regulate p53 expression. Of 221 genes, 52 (24%) were enriched at least 2-fold in the Bi-miR-34a PD in HCT116 cells (S2A Fig and S3 Table). Moreover, mRNAs for 42 of the 221 network genes (19%) were down-regulated at least 20% by miR-34a overexpression in HCT116 cells by mRNA microarray. 40% of these down-regulated mRNAs were also pulled down with Bi-miR-34a (S3 Table). The enrichment of p53 network genes in the Bi-miR-34a PD was validated by qRT-PCR in independent Bi-miR-34a PDs for 12 randomly selected p53 network genes (S2B Fig). All 12 genes were enriched 3–46 fold. Thus miR-34a binds to a large proportion of p53 network mRNAs.

### Enforced expression of individual miR-34a resistant p53-inhibitors does not abrogate miR-34a-mediated p53 transcriptional activation

*MDM4*, an important inhibitor of p53 transactivation [20–22], was the top enriched p53 network gene (68-fold, S3 Table) and the fifth most enriched gene of 2416 Bi-miR-34a-binding mRNAs in HCT116 [19]. Four other p53 inhibitors, previously identified as miR-34a targets [11, 13–15], the deacetylases *SIRT1* and *HDAC1* [23–26], the NuRD protein *MTA2* [25], and the transcription factor *YY1*, which represses p53 transcriptional activity and enhances p53 degradation [27, 28], were also enriched. miR-34a overexpression in HCT116 cells down-regulated mRNAs of all these genes, except *HDAC1* (S2A Fig). These 5 mRNAs were enriched in the Bi-miR-34a PD in HCT116 cells at least 10-fold relative to Bi-miRNA-control by qRT-PCR (S3A Fig), confirming the microarray results. *MDM4* was the most enriched (111-fold). *MDM4* mRNA and protein were strongly suppressed by miR-34a overexpression (S3B Fig). We identified 2 previously reported MREs in the 3’UTR of *MDM4* [29], and 3 CDS MREs, of which one was previously described [15] (S4 Fig). miR-34a down-regulation of HA-tagged MDM4, expressed without its 3’UTR, was abrogated by silent mutations in the CDS MREs, demonstrating their functionality. Thus *MDM4* is directly regulated by miR-34a by binding to at least 2 3’UTR and 3 CDS MREs.

**Fig 3:**
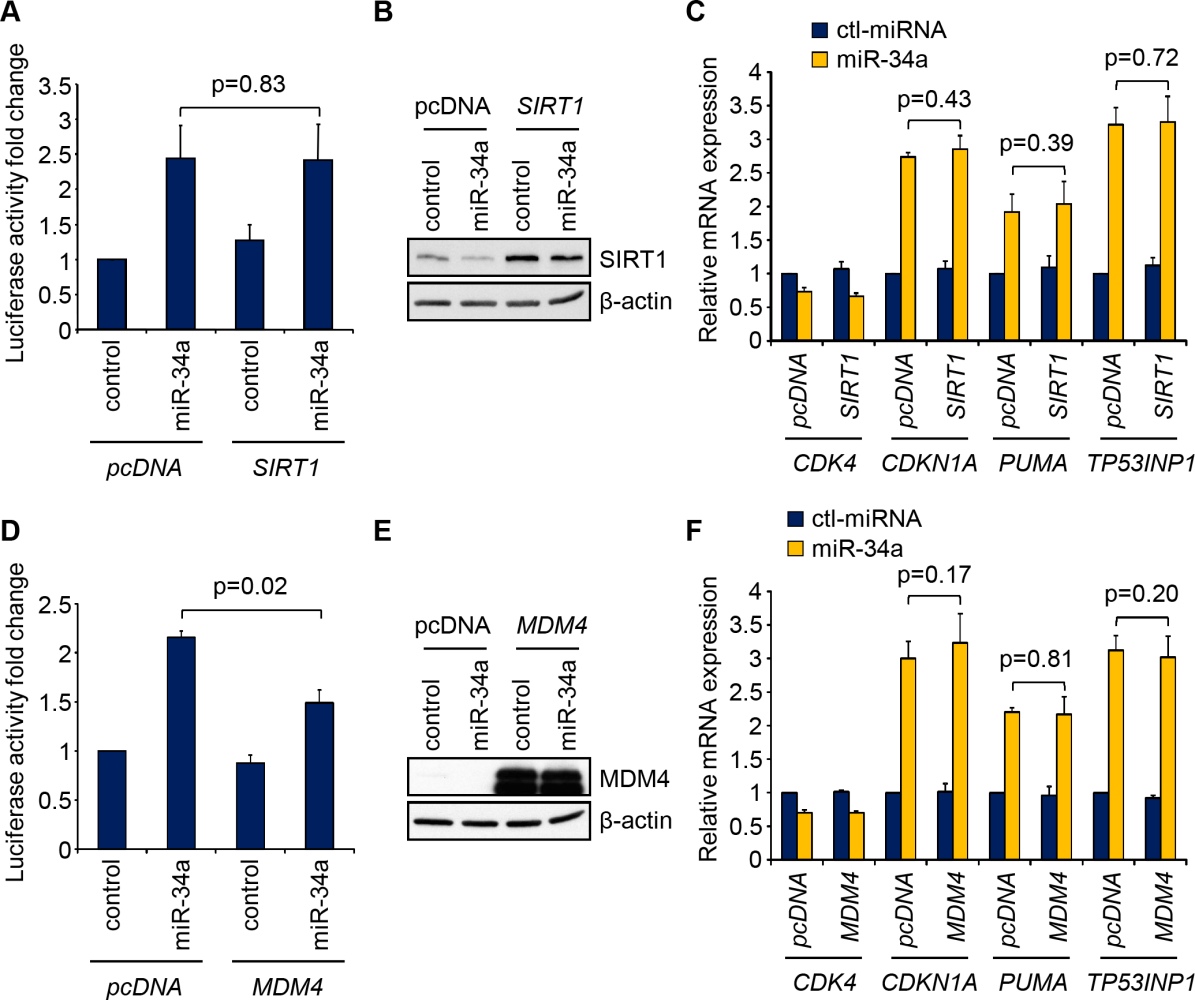
Overexpression of miR-34a-resistant *SIRT1* or *MDM4* does not inhibit miR-34a-mediated p53 transcriptional activation. **(A-C)** Overexpression of miR-34a resistant *SIRT1* (lacking its 3’UTR, immunoblot (**B**)) does not reduce the miR-34a-mediated increase in the activity of a luciferase reporter driven by a promoter containing 13 p53 consensus binding sites (pG13-luc) (**A**) or the miR-34a-mediated increase in mRNA of the p53 transcriptional targets *CDKN1A*, *PUMA* and *TP53INP1*, as measured by qRT-PCR (**C**). **(D-F)** Same analysis as in (**A-C**) performed after co-transfecting an HA-tagged *MDM4* gene with no 3’UTR and containing synonymous mutations of the miR-34a CDS MREs. All graphs show the mean +/- STDEV of at least three independent experiments (*, p<0.05; **, p<0.01, relative to control miRNA-transfected cells, by 2-tailed Student’s t-test).

To determine whether suppression of any p53 inhibitor has a predominant role in mediating miR-34a enhancement of p53 transcription, we evaluated the effect of co-transfecting miR-34a and individual miR-34a-resistant p53 inhibitor genes, lacking their 3’UTRs. Co-transfection of miR-34a-resistant *SIRT1*, which increased SIRT1 protein, did not significantly reduce miR-34a-induced pG13-luc promoter activity or increase p53 target (*CDKN1A*, *PUMA* and *TP53INP*) mRNAs (Fig 3A and 3C). Similar results were obtained after expressing miR-34a-resistant versions of *HDAC1*, *MTA2* and *YY1* (data not shown). Expression of miR-34a-resistant *MDM4* containing synonymous CDS-MRE mutations and lacking its 3’UTR significantly reduced miR-34a-enhanced pG13-luc reporter activity, but had no significant effect on *CDKN1A*, *PUMA* and *TP53INP1* mRNA levels (Fig 3D–3F). Thus, miR-34a suppression of multiple p53 inhibitors may be needed to increase p53 activity.

### *TP53* is a direct miR-34a target

**Fig 4:**
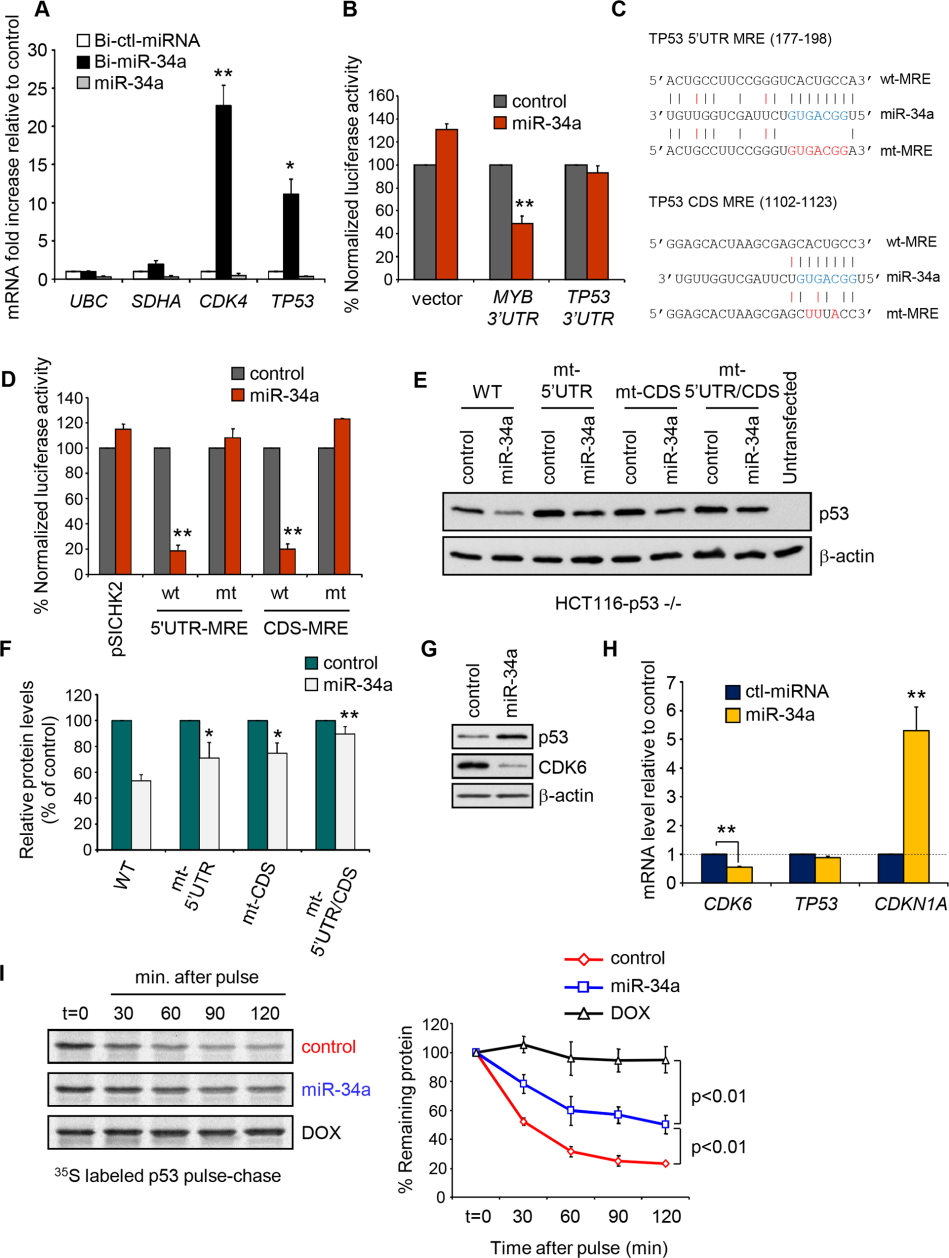
*TP53* is a direct miR-34a target. **(A)** *TP53* mRNA is enriched in Bi-miR-34a PDs in HCT116 cells. mRNA levels were determined by qRT-PCR and plotted as fold change relative to mRNAs pulled down with the Bi-control miRNA (Bi-ctl-miRNA). The housekeeping genes *UBC* and *SDHA* were used as negative controls. An additional control was PD of unbiotinylated miR-34a. **(B)** miR-34a does not affect the activity of a luciferase reporter containing the full length 3’UTR of *TP53*. A reporter containing the 3’UTR of *MYB* was used as positive control. Luciferase activity is relative to cells transfected with the control miRNA. **(C)** Pairing of miR-34a to predicted *TP53* MREs. The miR-34a seed region is highlighted in blue, while mutations (mt) introduced in the MREs are highlighted in red. Black dashes indicate Watson-Crick base pairing and red dashes G:U base pairing. The numbers in parentheses indicate the position of the MRE in the mRNA. **(D)** miR-34a recognition of predicted wild-type (wt) or mutant (mt) *TP53* miR-34a MREs cloned into the 3’UTR of *Renilla luciferase* were assessed in dual luciferase reporter assays in cells transfected with miR-34a relative to cells transfected with control miRNA. **(E,F)** The function of predicted *TP53* miR-34a MREs (wt or mt) in their native location in full length *TP53* mRNA was assessed in *TP53* ^*-/-*^ HCT116 cells cotransfected with control miRNA or miR-34a mimics and wt or mt p53 cDNA. p53 protein was analyzed by p53 vs β-actin immunoblot 48 hr later. A representative blot is shown in **(E)** and densitometry of p53 relative to β-actin signal in 3 independent experiments in cells transfected with miR-34a relative to cells transfected with control miRNA is shown in **(F)**. **(G)** miR-34a over-expression in p53-proficient HCT116 cells increases p53 protein. WT HCT116 cells were transfected with control or miR-34a mimics and protein levels were analyzed by immunoblot 48 hr post-transfection. CDK6 and β-actin immunoblots are shown as controls. **(H)** qRT-PCR analysis of *CDK6*, *TP53* and *CDKN1A* mRNA in samples from **(G)**. Levels are normalized to expression in control (ctl) miRNA-transfected cells. **(I)** miR-34a overexpression in HCT116 cells increases p53 protein stability. Pulse-chase analysis of p53 protein in HCT116 cells transfected with control or miR-34a mimics. DOX-treated HCT116 cells are a positive control. All graphs show the mean +/- STDEV of at least three independent experiments (*, p<0.05; **, p<0.01, relative to control miRNA-transfected cells, 2-tailed Student’s t-test). Immunoblots are representative of at least 3 independent experiments.

*TP53* mRNA was enriched 2.6-fold in the Bi-miR-34a PD in HCT116, suggesting it might be a miR-34a target (S2A Fig and S3 Table). This result was verified by qRT-PCR in independent PDs, where *TP53* mRNA was enriched 12-fold (Fig 4A). In comparison, *CDK4*, a well-known miR-34a target, was enriched 23-fold and *UBC* and *SDHA* housekeeping genes were not enriched. To determine whether *TP53* is a direct miR-34a target, we sought to identify MREs. When the *TP53* 3’UTR was cloned downstream of *Firefly luciferase*, miR-34a overexpression did not change luciferase activity (Fig 4B). Thus the *TP53* 3’UTR does not contain functional miR-34a MREs. *Rna22* [30] and PITA [31] identified potential MREs in the 5’UTR and CDS, respectively, each with perfect seed complementarity (Fig 4C). When these MREs were cloned downstream of the *Renilla luciferase* reporter, miR-34a overexpression strongly inhibited luciferase activity and seed-pairing mutations completely restored it (Fig 4D). Since MREs outside the 3’UTR often only weakly regulate gene expression, we next evaluated whether these MREs could mediate miR-34a inhibition in the full length mRNA. Co-transfection of *TP53* cDNA with miR-34a reduced p53 protein by 50% in p53-null HCT116 cells (Fig 4E and 4F). Importantly, synonymous mutations of one or both MREs partially or completely, respectively, restored p53 levels. Thus *TP53* is a miR-34a target gene that binds to miR-34a through noncanonical 5’UTR and CDS MREs.

Although *TP53* is a miR-34a target, miR-34a overexpression in p53-proficient HCT116 cells unexpectedly increased endogenous p53 protein in HCT116 cells (Fig 4G). This increase was not due to increased transcription (Fig 4H). As expected, miR-34 overexpression decreased the miR-34a target gene *CDK6* and increased the p53-activated gene *CDKN1A*, assessed as controls. To investigate why p53 protein increased, we analyzed p53 protein stability by pulse-chase analysis in control or miR-34a overexpressing HCT116 cells. miR-34a overexpression increased p53 half-life from ~30 to ~120 min (Fig 4I). As expected, p53 was stabilized in control cells treated with doxorubicin (DOX). Thus, miR-34a overexpression modulates p53 expression post-transcriptionally, both negatively and positively, via direct targeting of *TP53* and indirect enhancement of p53 stability.

### miR-34a effect on p53 depends on cellular context

**Fig 5:**
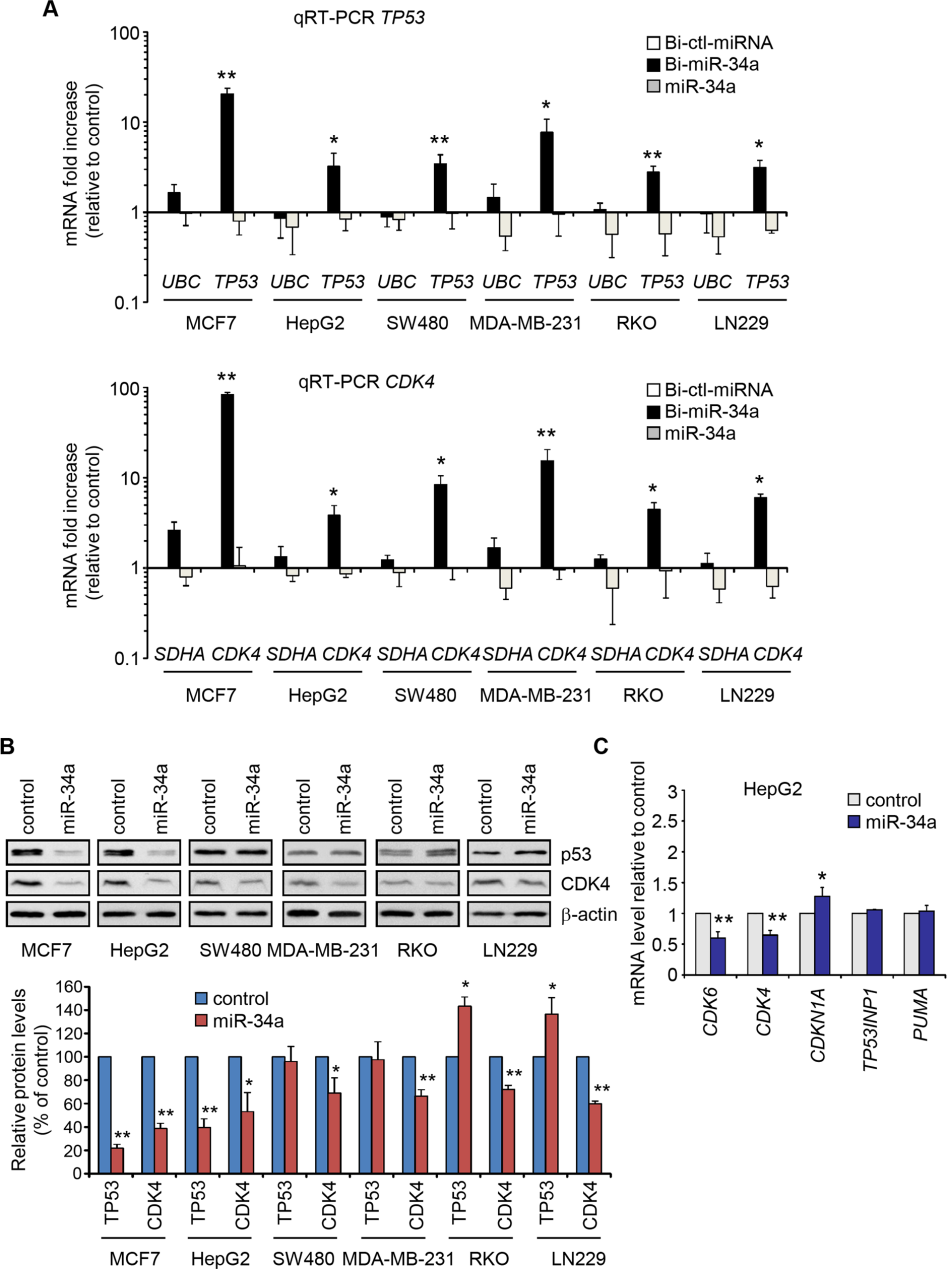
miR-34a overexpression differentially affects p53 levels in p53-sufficient cancer cell lines. **(A)** qRT-PCR analysis of the enrichment of *TP53* (top) and *CDK4* (bottom) mRNAs in Bi-miR-34a PDs in tumor cell lines of different origin. PD mRNA levels were plotted as fold change relative to mRNAs pulled down with the control Bi-miRNA (Bi-ctl-miRNA). The housekeeping genes *UBC* and *SDHA* served as negative controls. **(B)** Immunoblot of p53 and CDK4 in cells transfected with control or miR-34a mimics relative to β-actin (top). Protein levels were quantified by densitometry and the relative ratio of protein/β-actin was normalized to the value in cells transfected with control miRNA (bottom). **(C)** qRT-PCR analysis of p53 transcriptional target *CDKN1A*, *TP53INP1* and *PUMA* mRNAs after transfecting control or miR-34a mimics in HepG2 cells. mRNA levels of the miR-34a targets *CDK4* and *CDK6* are shown as controls. All experiments were performed at least 3 times and the graphs show mean +/- STDEV of replicate experiments (*, p<0.05; **, p<0.01, relative to control miRNA-transfected cells, 2-tailed Student’s t-test).

Because miR-34a can affect p53 expression by multiple mechanisms, we investigated the effect of miR-34a overexpression on p53 levels in a panel of tumor lines of different origin (MCF7 and MDA-MB-231 breast carcinomas, RKO and SW480 colorectal carcinomas, HepG2 hepatocellular carcinoma and LN229 glioblastoma). In all cells, Bi-miR-34a PD significantly enriched for *TP53* mRNA by 3-20-fold relative to Bi-miR-control PD (Fig 5A). The miR-34a target *CDK4* was also enriched by 3–80 fold. Housekeeping genes *UBC* and *SDHA* were not enriched and unbiotinylated miR-34a did not PD any genes (Fig 5A). However, although miR-34a overexpression significantly reduced CDK4 protein in all lines, it had a variable effect on endogenous p53 protein (Fig 5B). p53 significantly decreased in MCF7 and HepG2, increased in RKO and LN229, but was unchanged in SW480 and MDA-MB-231. Of note, miR-34a effect on p53 abundance was not related to p53 status since opposing effects were observed in MCF7/HepG2 vs. RKO/HCT116 cells, which all express wild-type (WT) p53. Consistent with the inhibitory effect of miR-34a on p53 protein in HepG2 cells, miR-34a overexpression in these cells only modestly induced expression of the p53 transcriptional target *CDKN1A*, and neither *TP53INP1* nor *PUMA* were induced (Fig 5C). This contrasts with the strong induction of p53-activated genes in HCT116 cells (Fig 1A). Thus, the effect of miR-34a overexpression on p53 levels and function depends on cellular context.

### miR-34a knockout does not inhibit the p53 response to genotoxic stress

**Fig 6:**
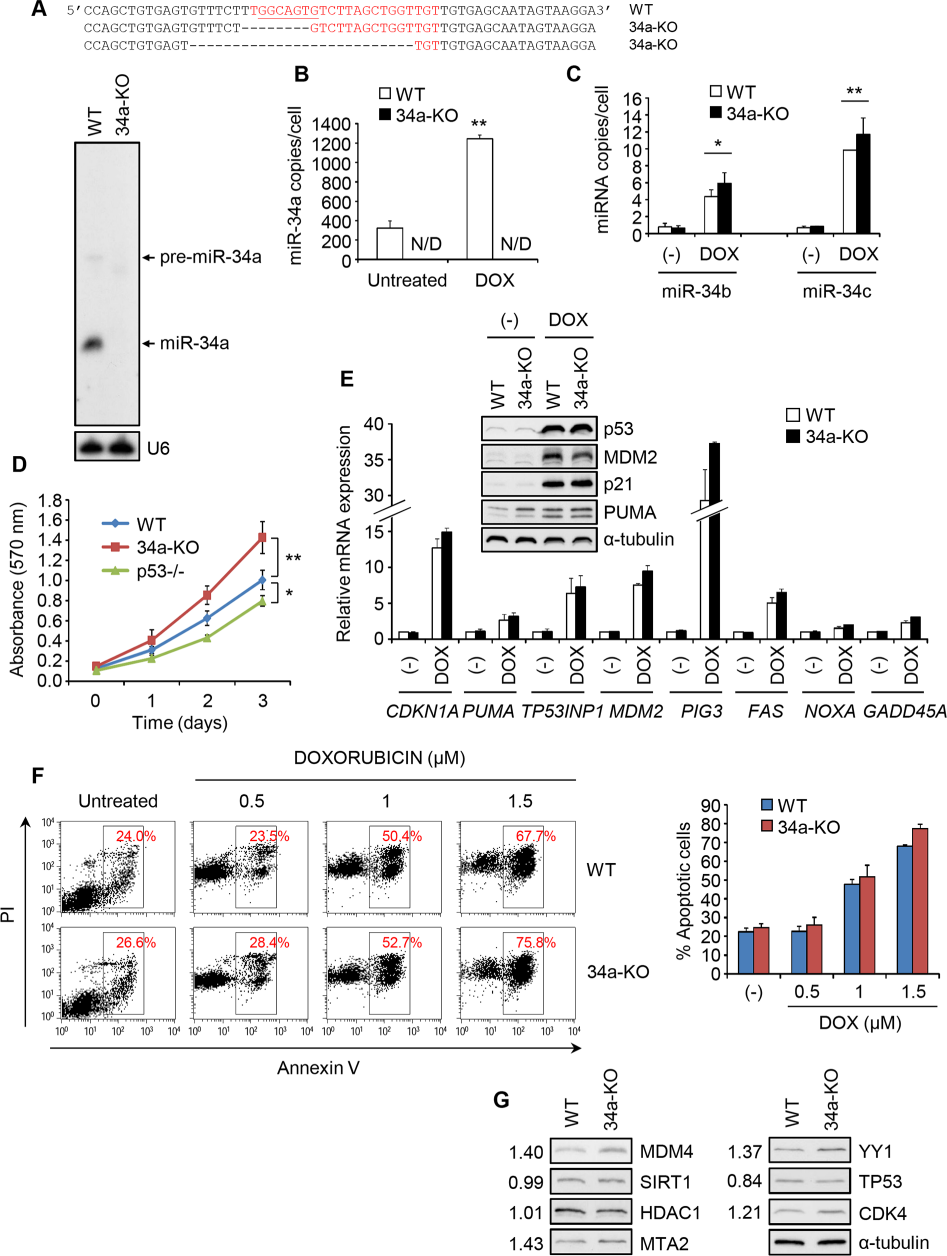
miR-34a-KO HCT116 cells have a normal p53 response to genotoxic stress. **(A)** Sequence of the miR-34a region in chromosome 1 in miR-34a-KO HCT116 cells compared to wild-type (WT) cells. The mature miR-34a sequence is in red, with the seed sequence underlined. The bottom panel shows the Northern blot for miR-34a in WT and 34a-KO HCT116 cells. U6 is a loading control. **(B)** miR-34a levels by qRT-PCR in WT or 34a-KO cells, untreated or DOX-treated for 16 hr. N/D, non-detectable. **(C)** Induction of miR-34b and miR-34c in WT and 34a-KO HCT116 cells after DOX treatment. Cells were treated with DOX as in (B). miR-34b/c levels were analyzed by qRT-PCR. **(D)** Proliferation of WT, p53-KO and 34a-KO cells by MTT cell proliferation assay. **(E)** qRT-PCR analysis of mRNA levels of p53 transcriptional targets in WT or 34a-KO HCT116 cells treated or not with DOX for 16 hr, relative to the untreated WT sample. The inset shows a representative immunoblot for p53 and some p53 transcriptional targets. α-tubulin is a loading control. **(F)** Apoptosis, assessed by annexin V/PI staining, in untreated or DOX-treated (48 hr) WT or 34a-KO HCT116 cells (DOX fluorescence in the PI channel increases “PI staining” in non-apoptotic cells). Representative dot plots are at left and the mean +/- STDEV of 3 independent experiments is at right. **(G)** Immunoblot of miR-34a target proteins in WT and 34a-KO HCT116 cells. The numbers on the left indicate the average signal intensity, normalized to the loading control, in 34a-KO/WT cells from 3 independent experiments. All experiments were performed at least 3 times and the graphs show the mean +/- STDEV from replicate experiments (*, p<0.05; **, p<0.01, relative to WT cells, 2-tailed Student’s t-test).

Because miRNA overexpression can exaggerate physiological importance, we next evaluated miRNA function by inhibiting the endogenous miRNA. Inhibition of miR34a using various antisense constructs did not affect the p53 response to DOX (data not shown). Because knockdown was incomplete, the lack of an effect could be due to residual miR-34a. We therefore deleted miR-34a in HCT116 cells using TALENs [32–34] (S5 Fig). miR-34a knockout (KO) HCT116 cells had deleted most of the seed region of miR-34a in one chromosome and most of the miR-34a sequence in the other, which abrogated miR-34a expression, by Northern blot and qRT-PCR (Fig 6A and 6B). In WT HCT116 cells, miR-34a is present at ~300 copies/cell under basal conditions and increases to ~1200 copies/cell after DOX. WT cells have <1 copy/cell of miR-34b and miR-34c, which only increases to 5 and 10 copies/cell, respectively, after DOX (Fig 6C). miR-34a KO did not lead to a significant compensatory increase in other family members under basal conditions or during stress. Because their levels are so low, endogenous miR-34b/c are unlikely to function in these cells.

miR-34a-KO cells proliferated at a faster rate than WT HCT116 cells, consistent with the known roles of miR-34a in inhibiting cell cycle progression and growth factor signaling [19, 35–38] (Fig 6D). p53-KO HCT116 cells proliferated more slowly than WT cells, suggesting that the increased proliferation of miR-34a-KO cells is p53-independent. After DOX treatment, induction of p53-regulated mRNAs and proteins and apoptosis, assessed by annexin V and propidium iodide (annexin-PI) staining, was comparable in miR-34a-KO and WT cells (Fig 6E and 6F). Similar results were obtained when 5-fluorouracil (5FU) was substituted for DOX (data not shown). miR-34a-KO cells showed no significant difference in p53 expression after DOX (Fig 6E) and only a moderate increase in abundance of the p53 inhibitors MDM4, MTA2 and YY1, but no change in SIRT1 or HDAC1 (Fig 6G). Thus the p53 response to genotoxic stress was unimpaired in HCT116 cells deficient in miR-34a.

**Fig 7:**
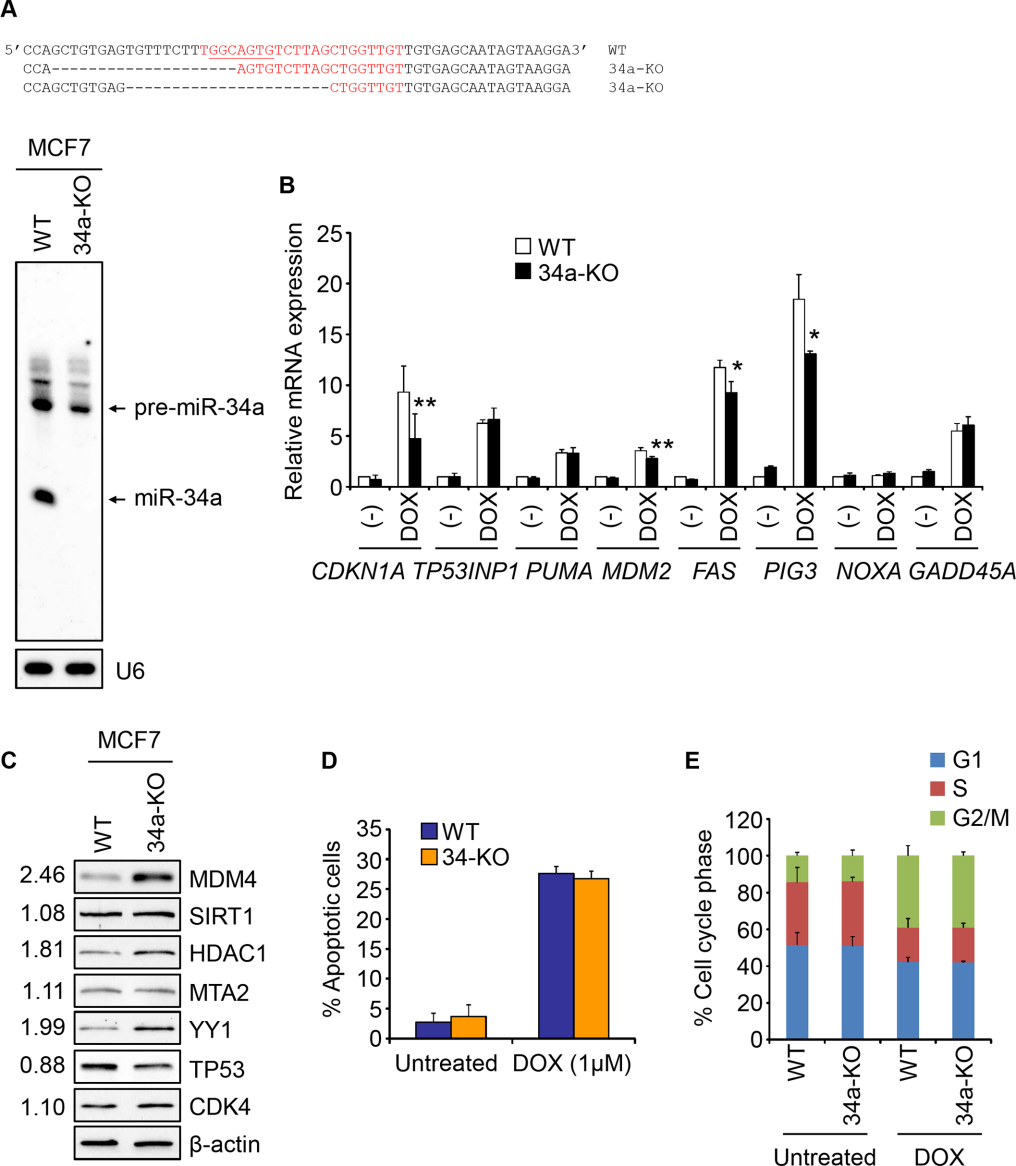
Unimpaired p53 response in miR-34a-KO MCF7 cells. **(A)** Sequence of the miR-34a region in chromosome 1 in miR-34a-KO MCF7 cells compared to WT cells. The mature miR-34a sequence is in red, with the seed sequence underlined. The bottom panel shows the Northern blot for miR-34a in WT and 34a-KO MCF7 cells. U6 is a loading control. **(B)** qRT-PCR analysis of mRNA levels of p53 transcriptional targets in WT or 34a-KO MCF7 cells treated or not with DOX for 16 hr. The data are plotted as the fold change normalized to the untreated WT sample. **(C)** Immunoblot of miR-34a target proteins in WT and 34a-KO MCF7 cells. The numbers on the left indicate the average relative signal intensity, normalized to the loading control, in 34a-KO/WT cells from three independent experiments. **(D)** Apoptosis, assessed by annexin V/PI staining, in untreated or DOX-treated (48 hr) WT or 34a-KO MCF7 cells. **(E)** Cell cycle analysis of untreated or DOX-treated (48 hr) WT or 34a-KO MCF7 cells. All experiments were performed at least 3 times and the graphs show the mean +/- STDEV of replicate experiments (*, p<0.05; **, p<0.01, relative to WT cells, 2-tailed Student’s t-test).

To evaluate whether the lack of miR-34a KO effect on p53 function might be particular to HCT116, we generated miR-34a KO MCF7 cells (Fig 7A). Induction of 4 of 8 p53 transcriptional targets was modestly, but significantly, reduced after DOX treatment in MCF7 KO, compared to WT, cells, but the other 4 genes were unaltered (Fig 7B). HDAC1, YY1 and MDM4 were more abundant in miR-34a-KO than WT MCF7 cells (Fig 7C), which might have contributed. However, WT and miR-34a-KO MCF7 cells did not differ in p53-mediated apoptosis or cell cycle arrest after DOX (Fig 7D and 7E). Taken together, our data suggest that miR-34a is dispensable for the p53-mediated response in human cells.

## Discussion

**Fig 8:**
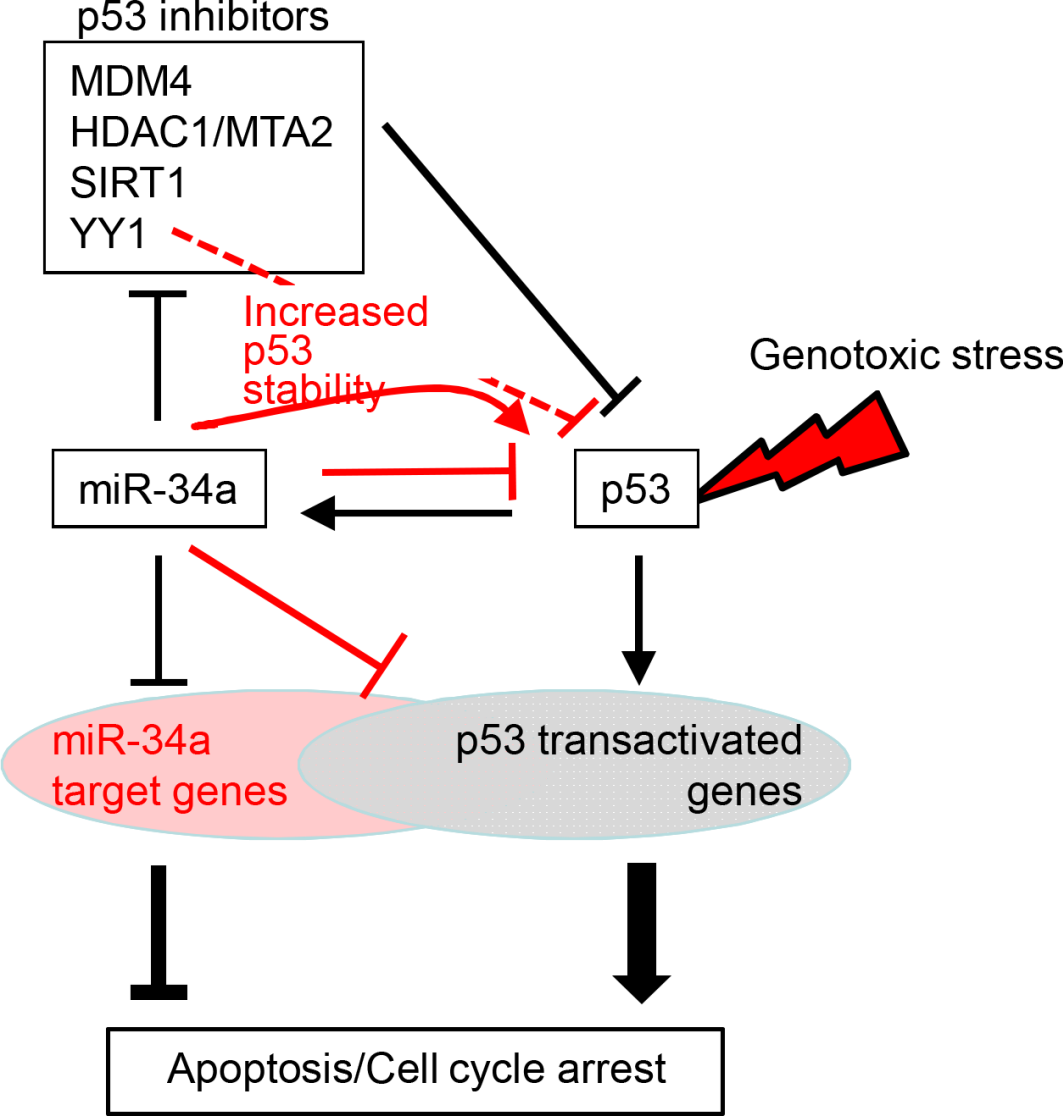
Model of miR-34a and p53 interactions. Competing negative and positive feedback loops determine the net effect of miR-34a on p53 function. Highlighted in red are the new layers of regulation revealed by our data. Activation of p53 by cellular stress leads to transcription of miR-34 miRNAs, which in turn can enhance p53 function by: (1) miR-34a-mediated inhibition of multiple negative regulators of p53 to further increase p53 transcriptional activity; and (2) miR-34a-mediated increase of p53 protein stability (miR-34a feed-forward loops); or inhibit p53 function by: (3) direct miR-34a-mediated inhibition of *TP53*; and (4) direct miR-34 inhibition of many p53-activated genes (negative feedback loops). The mechanism by which miR-34a increases p53 half-life is not known, but its suppression of *YY1*, whose gene product is known to enhance p53-MDM2 interactions may contribute. The net effect of miR-34a on the p53 response will depend on the relative importance of these pathways, which will be determined by differences in gene expression in each cell.

Since their initial identification as p53 transcriptional targets, the three members of the miR-34 family have been considered crucial mediators of the p53 response [39]. Here, we investigated in detail how the different miR-34 family members contribute to p53 function, the miR-34a targets that are relevant for its contribution and how much p53 relies on miR-34a. Our data confirm a feed forward loop in which p53 and miR-34a activate each other by p53 inducing miR-34a expression and miR-34a suppressing multiple p53 inhibitor genes. This positive effect is enhanced by an unexpected strong increase in p53 protein stability after over-expressing miR-34a. However, these positive feedback effects are counterbalanced by two important negative feedback mechanisms uncovered in this study – *TP53* is a direct target of miR-34a, recognized by noncanonical 5’UTR and CDS MREs, and about a quarter of p53-transactivated genes are also direct targets of miR-34a. These competing negative and positive feedback mechanisms counteract each other (Fig 8). As a consequence, knockout of miR-34a had no effect on the p53 response to genotoxic stress in two human cancer cell lines.

Previous reports have shown that miR-34a contributes to p53 function by targeting multiple p53 inhibitors, which include: proteins that deacetylate p53 (SIRT1, MTA2/HDAC1) [23–26], the negative transcriptional modulator MDM4 [20–22] and the transcription factor YY1 [27, 28]. Consistent with these reports all these genes were identified as hits in our Bi-miR-34a PD. MDM4, which together with MDM2 has the strongest effect on p53 function in knockout mice [40], is the strongest miR-34a target, containing at least 5 miR-34a MREs. We previously found that the extent of enrichment of a gene’s mRNA in the Bi-miR-34a PD correlates closely with how strongly its expression is suppressed [19]. MDM4 levels are likely tightly regulated by miR-34a; this target was one of the most highly enriched mRNAs in the Bi-miR-34a PDs and its mRNA and protein were strongly downregulated after miR-34a over-expression. However, expression of miR-34a-resistant versions of these genes individually, including *MDM4*, did not reduce the miR-34a-mediated increase of p53 transcriptional activity, suggesting that the coordinated knockdown of multiple inhibitor genes is needed to enhance p53 function. Our results are in conflict with previous experiments, which showed that enforced SIRT1 expression in HCT116 cells abrogated p53-dependent apoptosis induced by miR-34a [11]. Although the reason for this discrepancy is not clear, it is important to note that the reported difference in miR-34a-induced apoptosis between control and SIRT1 over-expressing HCT116 cells in that study was marginal (no more than 3%) [11].

An unexpected finding of this study was the weak effect of miR-34b or miR-34c over-expression on p53 function. Although mature miR-34b and miR-34c have sequences almost identical to miR-34a even outside the seed, over-expression of miR-34b or miR-34c, unlike over-expression of miR-34a, had little effect on p53 promoter activity and only weakly up-regulated the mRNA levels of p53 transcriptional targets. Although all three family members regulated cell cycle progression, miR-34b/c over-expression down-regulated mRNAs that mostly function in different biological processes than miR-34a. These data are consistent with a previous report showing differing proteomics profiles in HeLa cells over-expressing miR-34a or miR-34c [41]. Thus sequence determinants outside the seed might profoundly affect miR-34 family function by an unknown mechanism that is worth exploring. In mice, miR34b/c and the related miR-449 cluster are expressed specifically in multiciliated epithelia and their KO causes infertility and respiratory dysfunction [6, 7], supporting their distinct roles.

An important finding from our study is that *TP53* is a direct miR-34a target. *TP53* mRNA was highly enriched in the Bi-miR-34a PD. Two miR-34a MREs, located in the 5’UTR and coding region, mediated miR-34a inhibition of *TP53* expression in luciferase reporter assays. More importantly, mutation of theses MREs in a full length p53 cDNA expression vector abrogated the decrease in p53 protein levels observed upon miR-34a co-transfection in p53 null HCT116 cells, indicating that these MREs are functional in situ despite their locations. Although *TP53* is a direct miR-34a target gene, miR-34a over-expression in p53-proficient cells had variable effects on p53 levels in 7 cancer cell lines, even though miR-34a pulled down *TP53* in all lines. In fact miR-34a overexpression increased p53 levels in p53-proficient WT HCT116 cells, which was due to a 4-fold longer protein half-life.

Protein levels are jointly determined by rates of translation and protein degradation. Under basal conditions, p53 is highly unstable and its stability is regulated by the ubiquitin ligase MDM2, which is not a miR-34a target. miR-34a overexpression strongly enhanced the half-life of p53 (4-fold), at least in HCT116 cells. The net effect of these competing effects – inhibition of p53 production by direct miRNA targeting and inhibition of protein degradation indirectly by effects on other p53 network genes – could lead to increases or decreases in p53 level depending on their relative strengths in different contexts. We did not work out the mechanism behind increased p53 stability, but targeting of YY1, which promotes p53 ubiquitylation, might contribute [27, 28].

We used TALENs to generate cell lines in which the seed region of miR-34a was deleted in both chromosomes to completely eliminate production of the mature miRNA. miR-34a KO HCT116 cells showed a normal p53 response to stress. Although MDM4, MTA2 and YY1 protein levels were somewhat increased in 34a-KO cells as compared to WT, no significant difference was observed in the p53-mediated transcriptional response or in p53-induced apoptosis between WT and 34a-KO HCT116 cells after treatment with doxorubicin or 5FU. In contrast to our data, two previous reports described inhibition of p53-mediated apoptosis using antisense oligonucleotides against miR-34a in HCT116 and U2OS cells treated with 5FU or etoposide respectively [10, 11]. This discrepancy in HCT116 cells might be explained by potential off-target effects of the miR-34a antagonists. It is important to note that the effect of knocking out miR-34a on the response to genotoxic stress might differ in different cancer cell lines. For example HCT116 cells are deficient in mismatch repair because of mutations in hMLH1, which leads to genomic instability and increased resistance to some chemotherapy drugs [42]. However, additional analysis of the p53-mediated response to stress in 34a-KO MCF7 cells were consistent with those performed in HCT116 cells, suggesting that, although miR-34a can contribute to p53 function, its contribution is not essential for the p53-mediated response to stress. In all, our data suggest that the role of miR-34a in the p53 response in human cells, integrating the positive and negative effects on p53 network genes, is to stabilize and reinforce the p53 response, rather than promote it.

Our results showing that miR-34a is not essential for the p53 mediated response to stress are in agreement with data published by Concepcion et al reporting intact p53 function in miR-34 deficient mice [12]. The lack of a p53-related phenotype of miR-34a deficient cells might be explained by the high degree of complexity and redundancy that characterizes the p53 gene regulatory network [43]. Downstream p53 signals can be modulated by multiple factors at the transcriptional, post-transcriptional and post-translational levels [44]. These secondary gene regulatory networks presumably overlap with the gene network regulated by miR-34a. The net null effect of miR-34a on the p53 response may result from opposing effects of (1) enhancing p53 transcriptional activity by inhibiting its regulators, (2) suppressing the expression of p53 itself and many p53-activated genes, and (3) enhancing p53 stability. The sum of these positive and negative effects may reinforce the cell’s response to genotoxic damage without perturbing the net effect under most conditions. In addition, the lack of a strong effect of genetic deletion of miR-34a could also be secondary to functional redundancy provided by the other miR-34 members or other p53-regulated tumor suppressor miRNAs [45–49] or by the p53-independent miR-449 family, which shares a seed sequence with miR-34 [50].

Our analysis of miR-34a deficient cells focused on the p53-mediated response to stress. miR-34a function might be more critical in other p53-dependent processes, such as somatic cell reprogramming or inhibition of EMT (epithelial-mesenchymal transition) and metastasis [51–56]. In this regard, it has been shown recently that somatic cells from miR-34 deficient mice can be reprogrammed more efficiently [51]. In addition, a recent study suggests that miR-34a deficiency promotes tumorigenesis only when p53 is haploinsufficient [29]. miR-34a might be a critical regulator of other non-p53 related biological processes, which might differ depending on the cellular context. Future experiments with miR-34-deficient human cells should address the contribution of miR-34 in these other scenarios.

A better knowledge of the mutual functional dependence between miR-34 and p53 will help to understand miR-34 tumor suppressor function. Although miR-34a is well known for being a “p53 helper” miRNA [39], miR-34a expression is also induced independently of p53 [57–59]. Our previous global transcriptome analysis of miR-34a targets using Bi-miR-34a pull-downs suggested that miR-34a acts as a cellular brake in the proliferative and pro-survival response to growth factor stimulation [19]. Consistent with this idea, 34a-KO HCT116 cells and mouse KO cells proliferate more than WT cells. The p53-independent functions of miR-34a may turn out to be more important in vivo than its p53 effects. In support of this idea, miR-34a KO mice exhibit elevated bone resorption and reduced bone mass [60].

Because of its role in the p53 pathway and as a tumor suppressor, miR-34a mimics incorporated into liposomes are currently being evaluated for treatment of primary and metastatic liver cancers [61]. Our study suggests that the role of miR-34a in cancer is complex. It may be difficult to predict the antitumor effect of miR-34a, which may depend on the p53 status of the tumor and other tumor-specific genetic and epigenetic changes.

## Materials and Methods

### Cell culture and reagents

HCT116, MCF7, HepG2, SW480, MDA-MB-231, RKO and LN229 cells (ATCC) and HCT116-*TP53* ^-/-^ cells (Bert Vogelstein, Johns Hopkins University, Baltimore, MD) were maintained in DMEM supplemented with 10% fetal bovine serum, penicillin, streptomycin, HEPES and L-glutamine. DOX (Sigma, St. Louis, MO) was used at 0.34 μM unless otherwise indicated.

### miRNA mimics and transfections

miRNAs were from Dharmacon (GE Healthcare). 100 pmol miRNA was used to transfect 2x10^6^ cells by Amaxa nucleofection (Lonza).

### Plasmids and luciferase assays

Plasmids are listed in S4 Table. *CDKN1A*, *PUMA* and *BAX* promoters were previously described [62–64]. Luciferase assays used the Dual Luciferase Assay System (Promega) and a Synergy 2 Microplate Reader (Biotek) as described [59].

### Proliferation, apoptosis and cell cycle analysis

Proliferation was measured in quadruplicate by MTT Cell Proliferation Assay (ATCC) following the manufacturer’s protocol. Apoptosis was measured by flow cytometry after staining with Annexin V-APC (Life Technologies) and PI (Sigma) in 10 mM HEPES at pH 7.4, 140 mM NaCl, 2.5 mM CaCl_2_. For cell cycle analysis, cells were washed once in PBS, fixed in 70% ethanol at 4°C, washed again in PBS and resuspended in 0.1% (v/v) Triton X-100 in PBS, containing 200 μg/ml DNase-free RNase A and 20 μg/ml PI. Samples were incubated at 37°C for 15 min before analysis on a Becton-Dickinson FACScan using FlowJo (Tree Star, Inc).

### miRNA-Biotin pull-downs

Biotin PDs were performed as described [19]. mRNA in the Bi-miR-34a or Bi-cel-miR-67 control PD and input samples were quantified by microarray and qRT-PCR. The enrichment ratio is the ratio of mRNA in the Bi-miR-34a PD relative to the control PD, normalized to input levels.

### Immunoblot

Cells were lysed in RIPA buffer (50 mM Tris-HCl pH 7.4, 150 mM NaCl, 1 mM EDTA, 1% Triton X-100, 1% sodium deoxycholate, 0.1% SDS) supplemented with 1 mM PMSF, 1 μg/ml aprotinin, 1 μg/ml leupeptin. Protein concentration was measured by BCA Protein Assay (Pierce). Total protein (10 μg) was resolved on 10% SDS-PAGE, transferred to PVDF membranes (Immobilon-P, Millipore), and probed with primary antibodies against MTA2 (sc-55566), HDAC1 (sc-7872), SIRT1 (sc-15404) and p53 (sc-126) (Santa Cruz); YY1 (#2185), p21 (#2946), PUMA (#4976), CDK4 (#2906), CDK6 (#3136) and FAS (#8023) (Cell Signaling); MDM4 (mAb 8C6) and MDM2 (mAb 2A10) (EMD Bioscience); or anti-HA (clone 3F10, Roche), followed by incubation with horseradish peroxidase-(HRP) conjugated sheep anti-mouse or anti-rabbit Ig (Amersham). Signals were developed using SuperSignal West Femto (Pierce). Membranes were stripped and reprobed using α-tubulin mouse mAb (B-5-1-2, Sigma) or β-actin (JLA20, Millipore). Immunoblots were quantified by densitometry using VersaDoc MP 4000 (Bio-Rad).

### Pulse-chase analysis of p53 stability

p53 half-life was analyzed by p53 immunoprecipitation of ^35^S-labeled cellular extracts. Cells grown for 30 min at 37°C in DMEM without Met or Cys (Sigma), supplemented with 5% dialyzed FBS, were labeled for 30 min at 37°C with 150 μCi ^35^S/ml (EXPRE^35^S^35^S Protein Labeling Mix, PerkinElmer). Washed cells, incubated in chase medium (DMEM/10% FBS supplemented with unlabeled Met and Cys), were lysed with RIPA buffer. p53 Ab (2 μg/sample) was added and incubated overnight at 4°C, before adding 20 μl of protein A-sepharose (Santa Cruz sc-2001) for 2 hr. Immunoprecipitates were centrifuged, washed with lysis buffer, resuspended in SDS sample buffer and resolved by 10% SDS-PAGE. Fluorography used EN3HANCE (PerkinElmer), following the manufacturer’s instructions.

### RNA extraction, Northern blot and qRT-PCR

Total RNA was isolated using Trizol (Invitrogen). Northern blot analysis was previously described [59]. For qRT-PCR, cDNA was generated from 500 ng of total RNA using random hexamers (IDT) and Superscript III reverse transcriptase (Invitrogen). qRT-PCR was performed in triplicate using SYBR Green FastMix (Quanta) on a BioRad CFX96 with qRT-PCR primers from PrimerBank [65] (S5 Table). mRNA levels, normalized to *GAPDH*, were calculated using the 2^-ΔΔCT^ method. miRNA levels were quantified in triplicate using the TaqMan MicroRNA Assay (Applied Biosystems) per manufacturer’s instructions and normalized to U6. When calculating absolute miRNA copy numbers the Ct values obtained using the TaqMan MicroRNA Assay (Applied Biosystems), from equal amounts of total RNA, were used to calculate copies per μg of RNA by extrapolating the copy number from standard curves performed with known amounts of a miRNA RNA oligo (1 to 10^8^ copies). miRNA copies per μg was converted to copies per cell assuming 20 pg of total RNA per cell.

### miR-34a KO cells

miR-34a-KO cells were generated using TALENs (Transcription activator-like effector nucleases) targeting the miRNA seed (S5 Fig). miR-34a targeting TALENs were generated using the TALE Toolbox kit (Addgene cat#1000000019) [66]. Cells, transfected with 2 μg of each TALEN using Lipofectamine 2000 (Life Technologies), were plated by limiting dilution in 10 cm petri dishes 48 hr post-transfection. Single colonies were tested for miR-34 expression by qRT-PCR and negative colonies were verified by sequencing.

### Statistics

Data were analyzed by 2-sided student’s t-test. P-values <0.05 were considered significant.

### Microarray analysis

Microarray experiments were performed by the Microarray Core Facility of the Molecular Genetics Core Facility at Boston Children’s Hospital (supported by NIH-P50-NS40828 and NIH-P30-HD18655) using Illumina Human-HT12 BeadChips. Data, from three independent biological replicates, were analyzed using GenomeStudio Software (Illumina). Gene Ontology (GO) analysis was performed using DAVID [17, 18]. p53 network genes were compiled from the p53 Knowledgebase (http://p53.bii.a-star.edu.sg/index.php). Pathway analysis was performed using Ingenuity Pathway Analysis (IPA, Ingenuity Systems).

## Supporting Information

S1 Fig Analysis of miR-34 levels in miR-34 over-expressing samples. miR-34 levels in transfected samples from Fig 1D **(A)** and Fig 2 **(B)**, analyzed by qRT-PCR. Mean +/- SD of three independent experiments is shown in cells transfected with miR-34 family or cel-miR-67 (M-control) mimics. Copies/cell were calculated based on a standard curve. Of note, for both experiments, miR-34c expression is ~ 9 fold less than in miR-34b transfected samples. However, miR-34c is still highly expressed, even compared to DOX-treated HCT116-WT cells. miR-34c is increased in miR-34c transfected samples by 100X and 285X, relative to DOX treated HCT116-WT cells, respectively (compare S1A and S1B Fig to Fig 6C).
(TIF)

S2 Fig Multiple p53 inhibitors are direct targets of miR-34a. **(A)** Interactome (Ingenuity) of p53 network genes whose mRNAs were enriched at least 2-fold in the streptavidin PD of Bi-miR-34a relative to Bi-cel-miR-67 control PD in HCT116 cells. Highlighted in red are genes that were also significantly down-regulated in the gene microarray analysis of miR-34a over-expressing HCT116 cells. Genes highlighted in yellow indicate p53 transcriptional targets. These data were extracted from ^19^. **(B)** Validation of the gene microarray data in **(A)** in independent Bi-miR-34a PD experiments performed in HCT116 cells for 12 randomly selected genes. miR-34a PD mRNA levels were determined by qRT-PCR and plotted as fold change relative to mRNAs pulled down with the control Bi-miRNA (Bi-ctl-miRNA). PD after transfection of unbiotinylated miR-34a was another control. The housekeeping gene *UBC* was used as negative control. The bar graph shows the mean +/- STDEV of at least three independent experiments (*, p<0.05; **, p<0.01, relative to control miRNA-transfected cells, 2-tailed Student’s t-test).
(TIF)

S3 Fig MDM4, an important inhibitor of p53, is the top enriched p53 network gene (A) Enrichment of mRNAs for 5 p53 inhibitor genes and the housekeeping gene *SDHA* in the Bi-miR-34a PD relative to control-miRNA (Bi-ctl-miRNA) PD in HCT116 cells, assessed by qRT-PCR. Cells were also transfected with unbiotinylated miR-34a as a negative control. **(B)** Relative MDM4 mRNA (left) and protein (right) levels, assessed by qRT-PCR and immunoblot, respectively, in HCT116 cells transfected with miR-34a or control-miRNA (ctl-miRNA). The number indicates the % of remaining protein, normalized to β-actin, in 3 independent miR-34a overexpressing samples.
(TIF)

S4 Fig *MDM4* is a direct miR-34a target that contains multiple 3’UTR and CDS MREs. **(A)** Complementarity of miR-34a and validated *Rna22*-predicted MREs within the 3’UTR and CDS of *MDM4*. The miR-34a seed region is in blue, while mutations introduced in the MREs are highlighted in red. Black dashes indicate Watson-Crick base pairing and red dashes indicate G:U base pairing. The numbers in parenthesis indicate the position of the MRE in the mRNA. **(B)** Mutations in *MDM4* 3’UTR MREs 4 and 5 abrogate miR-34a inhibition of luciferase activity of a reporter containing a 1022 bp fragment of the *MDM4* 3’UTR. Dual luciferase activity was normalized to the value in control (ctl)-miRNA transfected cells. **(C)** Luciferase reporter assay of *Rna22*-predicted miR-34a CDS MREs of *MDM4* cloned into the 3’UTR of *Renilla luciferase*. AS-34a indicates a psiCHECK2 reporter containing a perfect match for miR-34a, used as positive control. Normalization as in (B). **(D)** Mutations in *MDM4* CDS MREs 1, 3 and 4 increase MDM4 protein after miR-34a transfection. The representative immunoblot (left) shows HA-tagged MDM4 in 293T cells co-transfected with a plasmid encoding for WT or mutated (mt) *HA-MDM4* and with control miRNA or miR-34a mimics. β-actin is a loading control. Protein levels were quantified by densitometry of independent experiments (right) and the relative ratio of MDM4-HA/β-actin was normalized to the value in cells transfected with control miRNA. All graphs show the mean +/- STDEV of at least three independent experiments (*, p<0.05; **, p<0.01, relative to control miRNA-transfected cells, 2-tailed Student’s t-test).
(TIF)

S5 Fig TALEN designs for targeted deletion of miR-34a miRNA. The figure shows the binding sites for each pair of TALENs, left (L) and right (R), targeting miR-34a miRNA (underlined). The DNA sequence corresponds to the miRNA genomic region. Highlighted in blue and red are the sequences that form the miRNA hairpin, with the mature miRNA sequence in red. The seed sequence is in light green and underlined. The complete TALEN target sequence is shown abbreviated (5’-TN^19^N^18^N^19^A-3’). The first base of the binding site, which is required to be a “T”, is highlighted in dark green.
(TIF)

S1 Table Genes down-regulated by miR-34 over-expression in HCT116 cells. (XLSX)

S2 Table Functional Annotation Analysis of downregulated genes in HCT116 cells overexpressing miR-34 using DAVID Bioinformatics tool. (XLSX)

S3 Table Analysis of p53 network genes, compiled from p53 Knowledgebase, in Biot-miR-34a pull-downs. (XLSX)

S4 Table List of plasmids used in the study. (XLSX)

S5 Table List of qRT-PCR primers. (XLSX)
