# Supplementary figures and images for: miR-34 and p53: New Insights into a Complex Functional Relationship
Source: PLoS One. 2015 Jul 15;10(7):e0132767. doi: 10.1371/journal.pone.0132767 (PMC4503669; doi:10.1371/journal.pone.0132767)

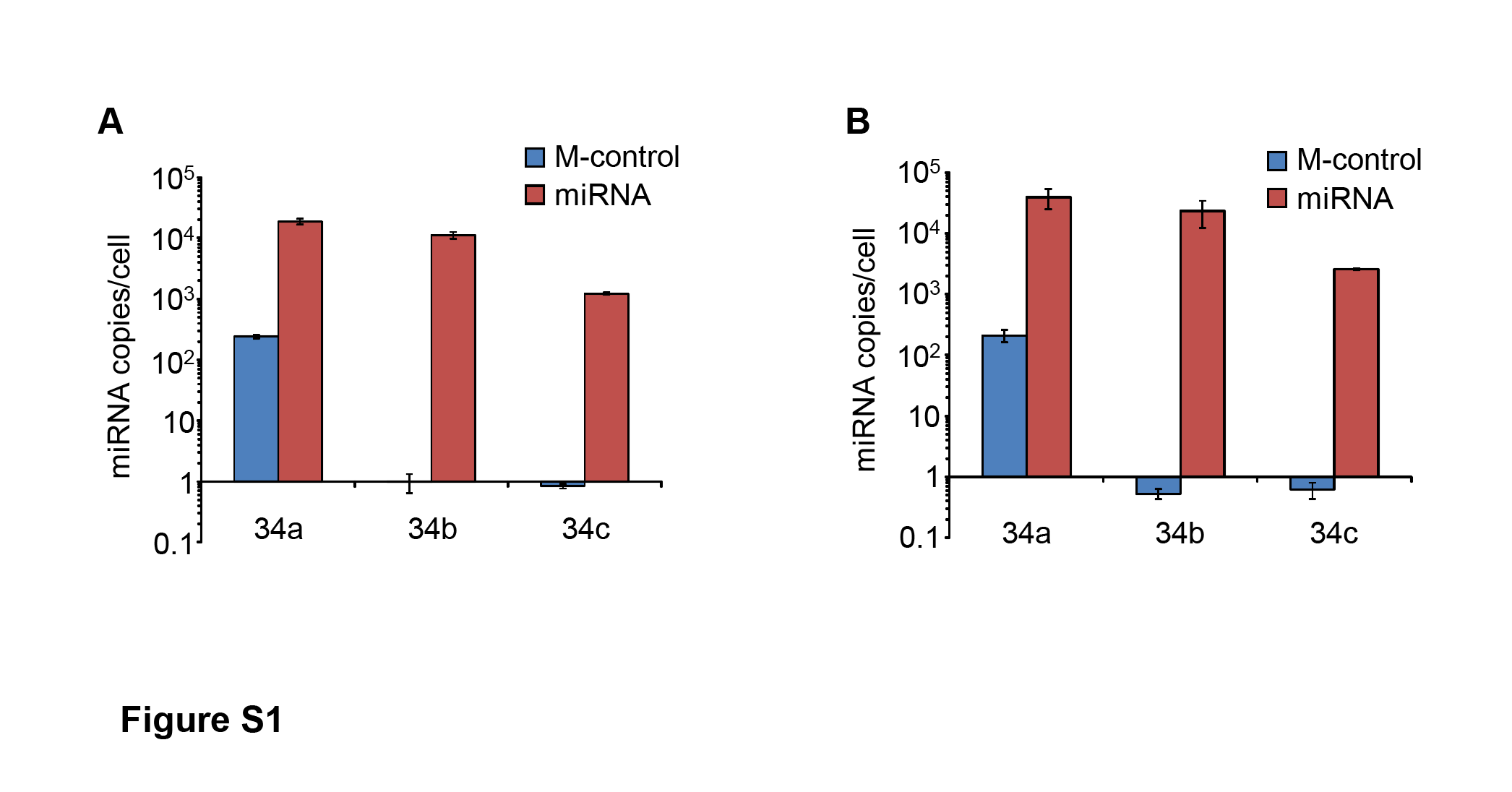

Supplement: S1 Fig — miR-34 levels in transfected samples from Fig 1D (A) and Fig 2 (B), analyzed by qRT-PCR. Mean +/- SD of three independent experiments is shown in cells transfected with miR-34 family or cel-miR-67 (M-control) mimics. Copies/cell were calculated based on a standard curve. Of note, for both experiments, miR-34c expression is ~ 9 fold less than in miR-34b transfected samples. However, miR-34c is still highly expressed, even compared to DOX-treated HCT116-WT cells. miR-34c is increased in miR-34c transfected samples by 100X and 285X, relative to DOX treated HCT116-WT cells, respectively (compare S1A and S1B Fig to Fig 6C). (TIF) [file pone.0132767.s001.tif]

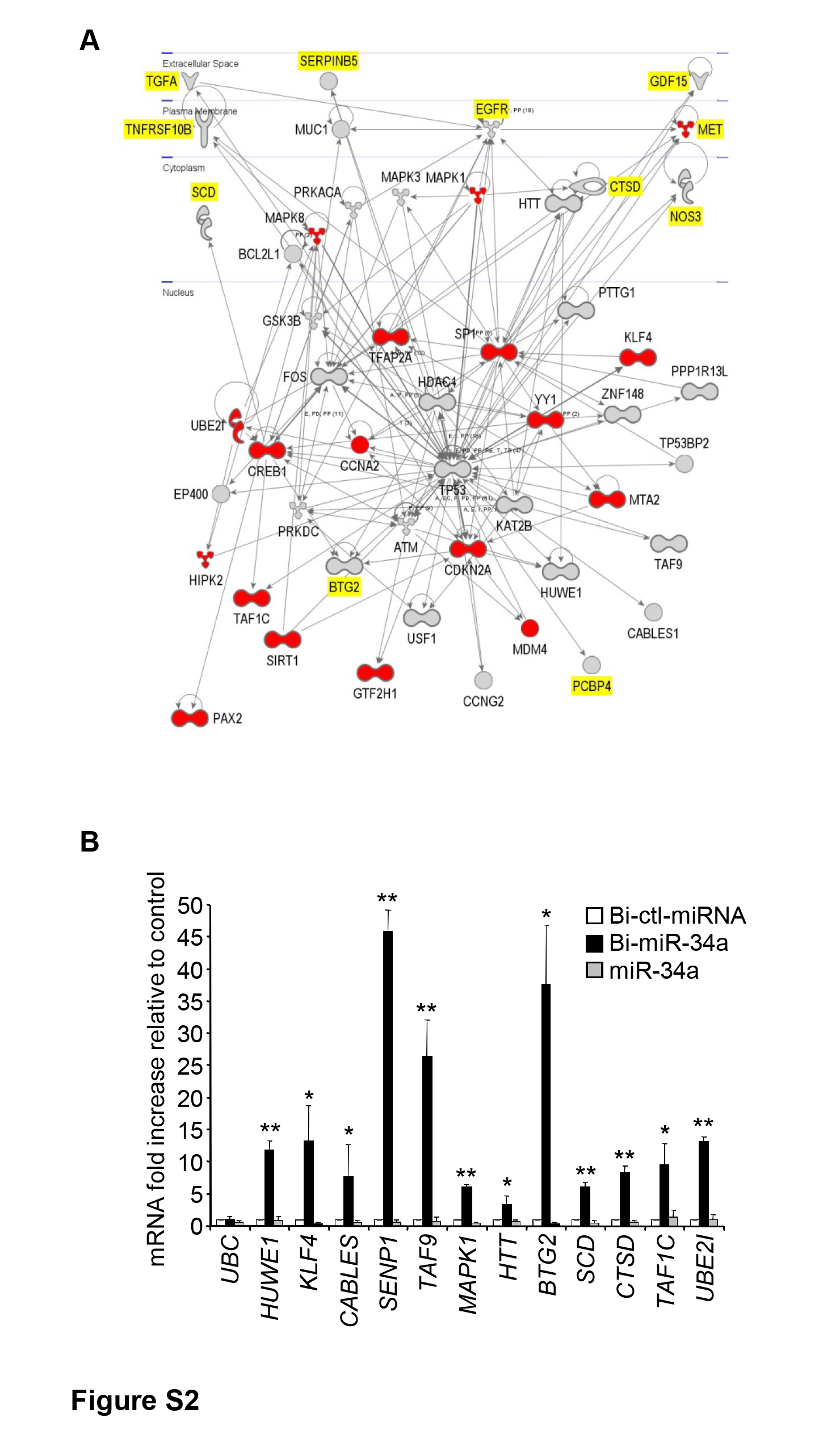

Supplement: S2 Fig — (A) Interactome (Ingenuity) of p53 network genes whose mRNAs were enriched at least 2-fold in the streptavidin PD of Bi-miR-34a relative to Bi-cel-miR-67 control PD in HCT116 cells. Highlighted in red are genes that were also significantly down-regulated in the gene microarray analysis of miR-34a over-expressing HCT116 cells. Genes highlighted in yellow indicate p53 transcriptional targets. These data were extracted from 19. (B) Validation of the gene microarray data in (A) in independent Bi-miR-34a PD experiments performed in HCT116 cells for 12 randomly selected genes. miR-34a PD mRNA levels were determined by qRT-PCR and plotted as fold change relative to mRNAs pulled down with the control Bi-miRNA (Bi-ctl-miRNA). PD after transfection of unbiotinylated miR-34a was another control. The housekeeping gene UBC was used as negative control. The bar graph shows the mean +/- STDEV of at least three independent experiments (*, p<0.05; **, p<0.01, relative to control miRNA-transfected cells, 2-tailed Student’s t-test). (TIF) [file pone.0132767.s002.tif]

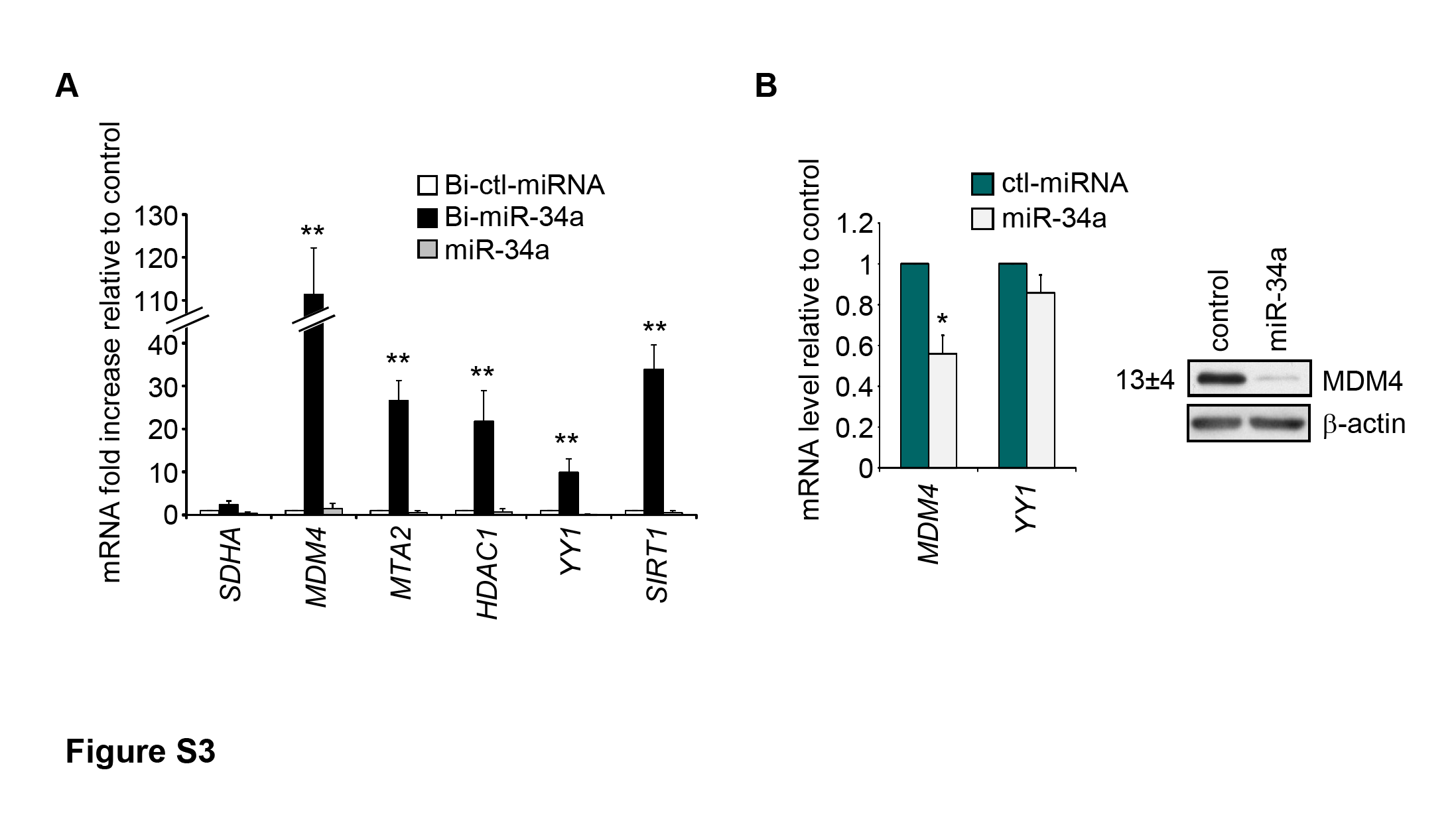

Supplement: S3 Fig — Cells were also transfected with unbiotinylated miR-34a as a negative control. (B) Relative MDM4 mRNA (left) and protein (right) levels, assessed by qRT-PCR and immunoblot, respectively, in HCT116 cells transfected with miR-34a or control-miRNA (ctl-miRNA). The number indicates the % of remaining protein, normalized to β-actin, in 3 independent miR-34a overexpressing samples. (TIF) [file pone.0132767.s003.tif]

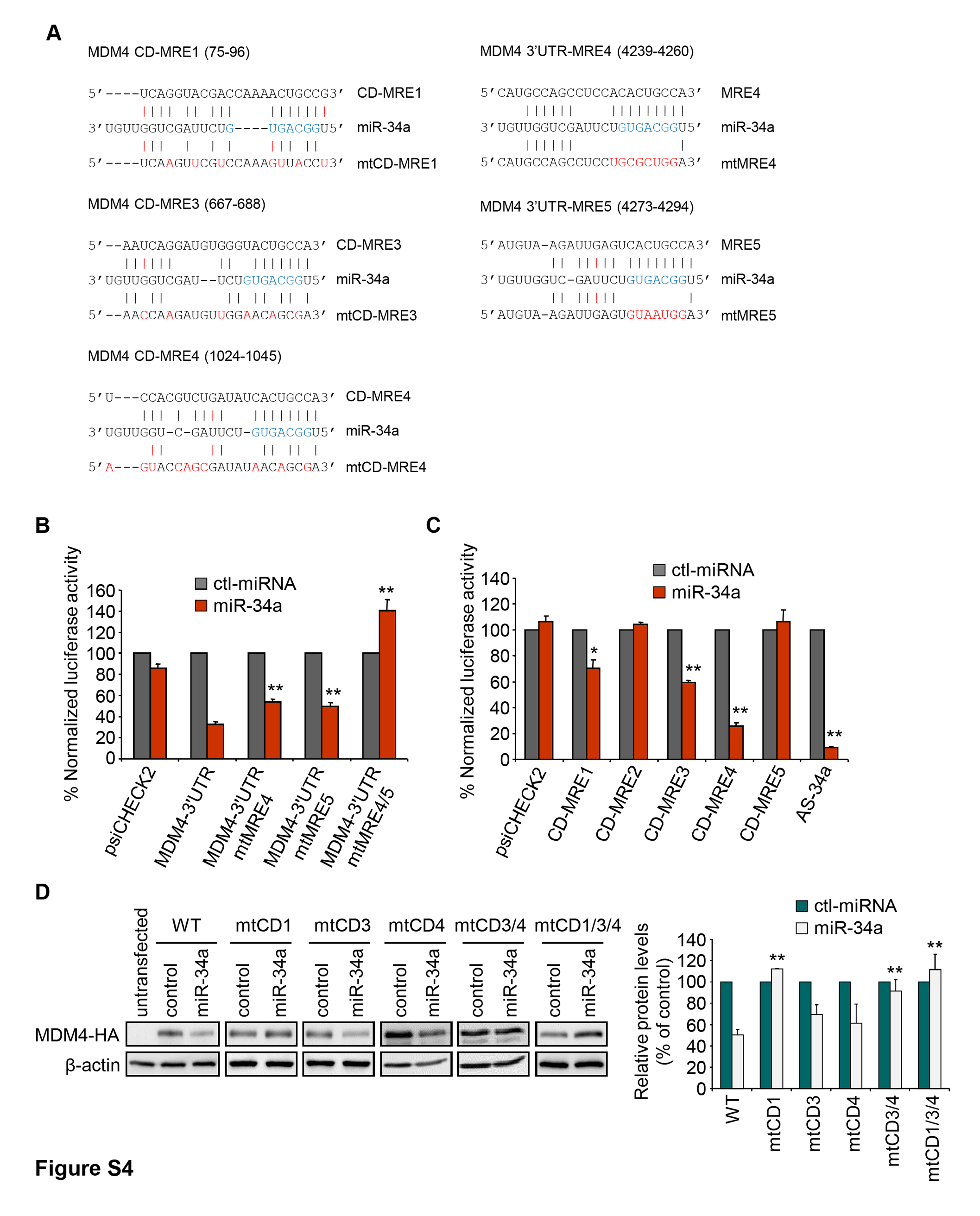

Supplement: S4 Fig — (A) Complementarity of miR-34a and validated Rna22-predicted MREs within the 3’UTR and CDS of MDM4. The miR-34a seed region is in blue, while mutations introduced in the MREs are highlighted in red. Black dashes indicate Watson-Crick base pairing and red dashes indicate G:U base pairing. The numbers in parenthesis indicate the position of the MRE in the mRNA. (B) Mutations in MDM4 3’UTR MREs 4 and 5 abrogate miR-34a inhibition of luciferase activity of a reporter containing a 1022 bp fragment of the MDM4 3’UTR. Dual luciferase activity was normalized to the value in control (ctl)-miRNA transfected cells. (C) Luciferase reporter assay of Rna22-predicted miR-34a CDS MREs of MDM4 cloned into the 3’UTR of Renilla luciferase. AS-34a indicates a psiCHECK2 reporter containing a perfect match for miR-34a, used as positive control. Normalization as in (B). (D) Mutations in MDM4 CDS MREs 1, 3 and 4 increase MDM4 protein after miR-34a transfection. The representative immunoblot (left) shows HA-tagged MDM4 in 293T cells co-transfected with a plasmid encoding for WT or mutated (mt) HA-MDM4 and with control miRNA or miR-34a mimics. β-actin is a loading control. Protein levels were quantified by densitometry of independent experiments (right) and the relative ratio of MDM4-HA/β-actin was normalized to the value in cells transfected with control miRNA. All graphs show the mean +/- STDEV of at least three independent experiments (*, p<0.05; **, p<0.01, relative to control miRNA-transfected cells, 2-tailed Student’s t-test). (TIF) [file pone.0132767.s004.tif]

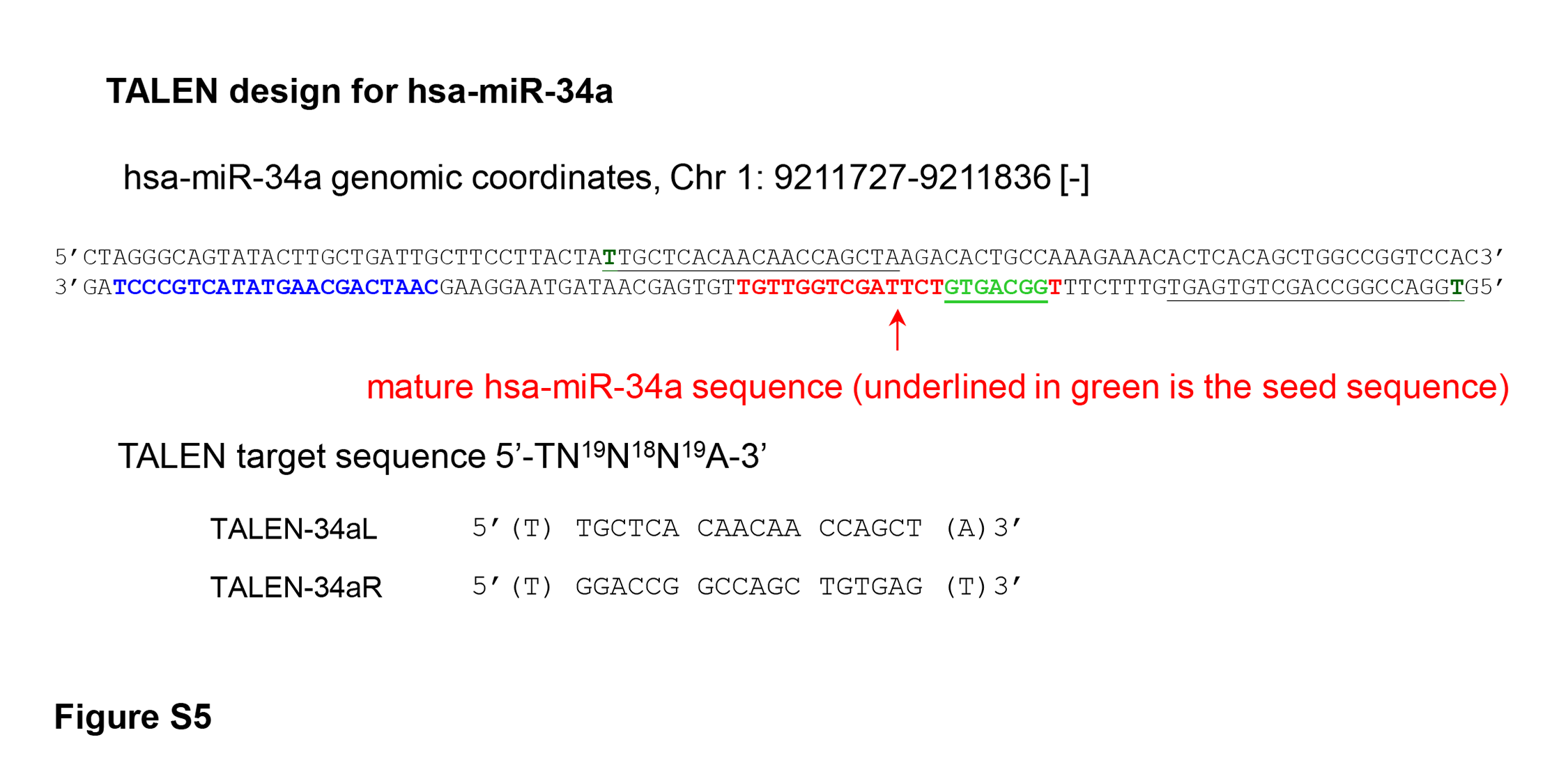

Supplement: S5 Fig — The figure shows the binding sites for each pair of TALENs, left (L) and right (R), targeting miR-34a miRNA (underlined). The DNA sequence corresponds to the miRNA genomic region. Highlighted in blue and red are the sequences that form the miRNA hairpin, with the mature miRNA sequence in red. The seed sequence is in light green and underlined. The complete TALEN target sequence is shown abbreviated (5’-TN19N18N19A-3’). The first base of the binding site, which is required to be a “T”, is highlighted in dark green. (TIF) [file pone.0132767.s005.tif]
